# A case of vulvar cutaneous endometriosis presenting as a chronic, fluctuating labial ulcer in an adolescent female

**DOI:** 10.1016/j.jdcr.2025.04.023

**Published:** 2025-05-02

**Authors:** Alisa A. Suen-Wallach, Angela J. Jiang, Erin L. Foster

**Affiliations:** a University of North Carolina at Chapel Hill, School of Medicine, Chapel Hill, North Carolina; b Department of Dermatology, Oregon Health and Science University, Portland, Oregon

**Keywords:** cutaneous endometriosis, genital, mucosal, mucosal ulcer, vulva, vulvar dermatoses, vulvar ulcer, vulvovaginal disease, women’s health

## Introduction

Endometriosis affects approximately 10% of reproductive-age females and is characterized by endometrial tissue found outside the uterine cavity.^1^^,^^2^ The ectopic endometrium responds to physiologic hormone fluctuations resulting in expansion, sloughing, and bleeding.^2^ Current leading theories for the pathogenesis of endometriosis include tissue arising from coelomic metaplasia, dysplastic migration during embryogenesis, or seeding from displaced endometrial tissue via retrograde menses, mechanical transplantation during surgery, or hematogenous spread.^2^, ^3^, ^4^

While endometriosis is most often localized to the peritoneal cavity and abdominopelvic organs, it has rarely been observed in the skin.^5^ Cutaneous endometriosis is most often associated with scarring, either postsurgical (eg incisions from cesarean section, laparotomy, or laparoscopy) or physiological (umbilicus not associated with umbilical laparoscopy incision). Few cases of vulvar cutaneous endometriosis have been reported in the literature, with lesions localized to the dermis or subcutis of the mons pubis, labia majora, perineum, or Bartholin glands.^4^^,^^6^

Here, we present a case of a 15-year-old female with cutaneous endometriosis on the vulva that demonstrated cyclical sloughing and ulceration during menses.

## Case report

The patient initially presented to the emergency department at age 14 with severe vulvar pain during menstruation. On exam, ulceration on the left labium minus was identified. The patient denied prior sexual activity, abuse or trauma to the area, changes in personal care products or undergarment materials. Menarche began at age 12, with primary dysmenorrhea, irregular cycles, and occasionally prolonged intermenstrual intervals. Neither the patient nor her parents were aware of any prior vulvar abnormalities or history of genital lesions, adhesions, or infections. Work-up included a computed tomography scan, which ruled out abscess or mass, and an infectious screening, including a negative HSV PCR.

One year later, the patient presented to the dermatology clinic for further evaluation. She reported constant, burning pain of the vulva that worsened with urination and sitting, with intermittent, unpredictable flares. Her past medical history included intermittent oral aphthous ulcers, primary dysmenorrhea managed with combined oral contraceptive pills, and trichotillosis affecting the scalp, eyelashes, and pubic hair.

**Fig 1:**
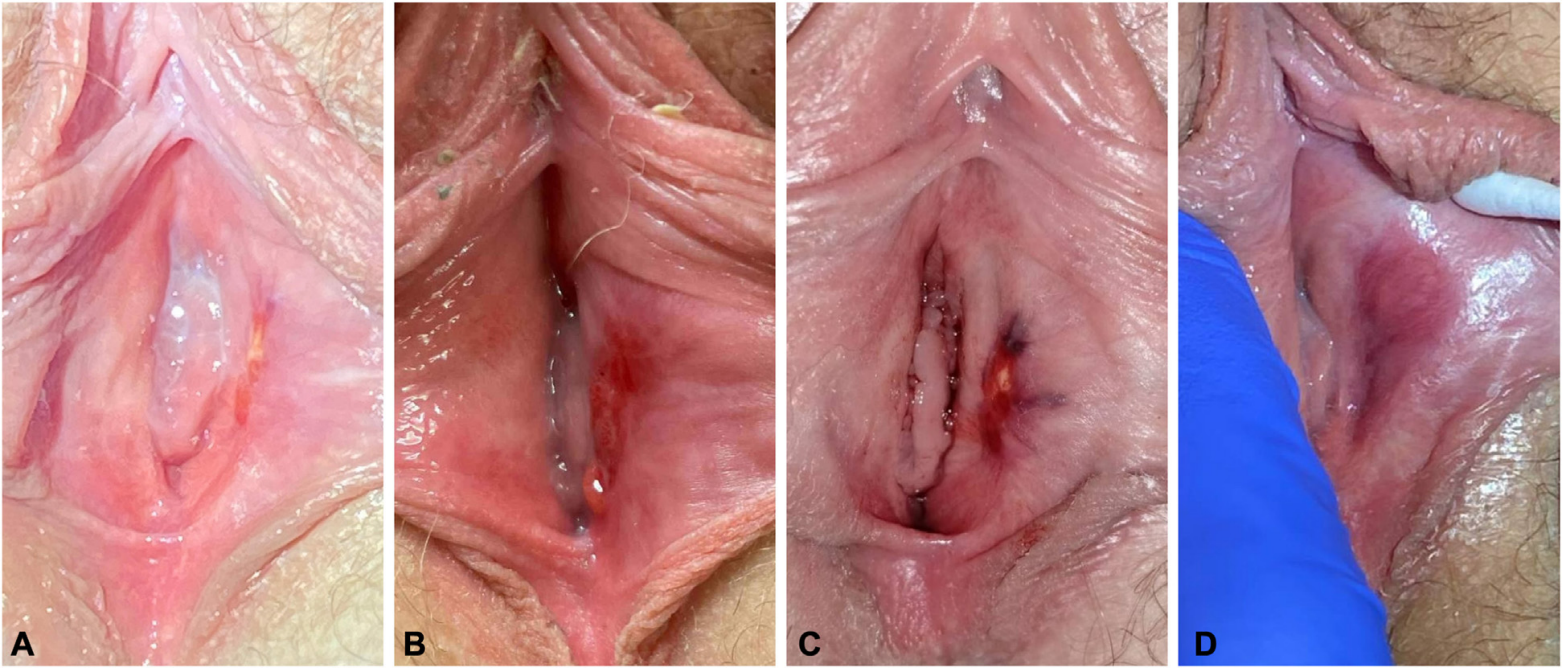
Morphology of the labial lesion throughout the menstrual cycle and post excision: **A,** A linear *pink* erosion with a central *yellow* base located on the left vestibule; intermenstrual phase. **B,** A superolateral red erosion on the medial labium minus extending to an inferomedial thin, macerated, red ulcer within the left vestibule with a smooth, domed, pink papule at the inferior edge; intermenstrual phase. **C,** Dark red, desquamating erosion with a central pink-yellow papule and peripheral petechiae located on the left vestibule; confirmed during menses. The surrounding epidermis appears contracted around the lesion. **D,** Left labium minus 2 months after surgical excision of the cutaneous endometriosis.

On physical exam, there was a 1.5 cm ulcer located on the medial surface of the left labium minus. On 3 subsequent exams, the lesion remained confined to the medial left labium minus, but the clinical appearance varied significantly (Fig 1, *A*-*C*). At the initial and subsequent visits, the patient and her parents requested empiric treatment without biopsy. Over the course of 12 months, the patient was trialed on topical and oral antibiotics, antivirals, oral prednisone, topical clobetasol, intravaginal hydrocortisone suppositories without resolution or substantial improvement. The only treatment that improved her pain was topical lidocaine 5% ointment.

**Fig 2:**
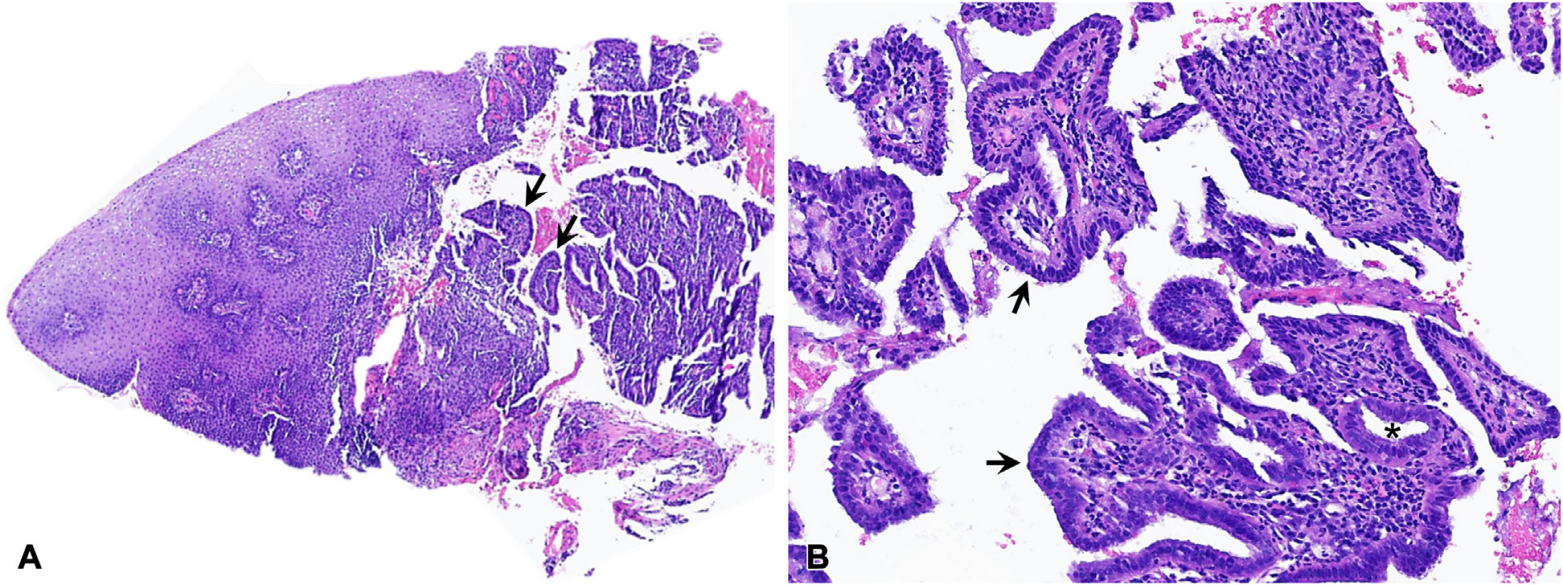
Histology of the vulvar biopsy: **A,** Nonkeratinized stratified squamous epithelium of the labial mucosa (*left*) adjacent to clusters of ductal epithelium (*right, arrows*) and surrounding hemorrhage; 40×. **B,** Aggregates of ductal epithelia (*arrows*) with surrounding stroma, admixed acute and chronic inflammation, and hemorrhage; endometrial gland (asterisk); 120×.

After iterative discussions with the patient and her parents, a 4-mm punch biopsy was performed at the periphery of the lesion. The biopsy showed fragmented aggregates of simple cuboidal to columnar epithelium adjacent to fibrotic, inflamed stroma with neutrophils, plasma cells, and extravasated erythrocytes (Fig 2). Immunohistochemical stains show PAX8 and estrogen receptor staining within epithelial cells, and estrogen receptor and CD10 staining in the adjacent stroma. Direct immunofluorescence studies were negative. These findings were consistent with cutaneous endometriosis. The biopsy had been taken at the end of the patient’s menses.

The patient requested surgical treatment, and the tissue was excised by gynecology under general anesthesia. This provided definitive treatment, complete resolution of symptoms and no local recurrence to date (Fig 1, *D*). She underwent intravaginal ultrasound with no other loci of endometriosis identified, and she continues to follow regularly with a gynecologist.

## Discussion

Endometriosis presenting as an isolated lesion on the mucosal surface of the labium minus is a rare manifestation. The delay in reaching a diagnosis stemmed from the unusual disease presentation and lack of histology. Skin biopsies in minors, especially on the genitals, are often avoided by patients, parents and clinicians, as in this case. Additionally, the adolescent patient’s irregular menstrual cycle made it challenging to correlate the intermittent worsening of the lesion with menses.

Diagnoses that were considered prior to biopsy included fixed drug eruption (culprit medications were stopped with no improvement), lichen planus (no other signs on complete mucosal and skin exam), and complex aphthosis (single vulvar ulcer did not resemble typical aphthous morphology). Other less likely etiologies were HSV (multiple negative PCRs, negative sexual history) or mucous membrane pemphigoid (highly unlikely due to young age). Although the patient suffered from trichotillosis involving her pubic hair, the labia minora lack hair and were not a target for her.

Given that the patient’s lesion was superficial with no deeper involvement and did not develop in the setting of known tissue trauma, infection, scarring, surgery, or pregnancy, its pathogenesis does not easily align with the typical categories for vulvar or cutaneous endometriosis. Translocation of viable endometrial tissue during menstruation and labial implantation—in line with the retrograde menstruation theory— seems like the most plausible mechanism.^7^ The timing of this event could have occurred during the 2 years since menstruation began or during vaginal bleeding in the postnatal “minipuberty,” attributed to withdrawal of placental hormones, with tissue remaining dormant until menarche.^8^

Surgical removal of endometriosis can offer definitive treatment with a low recurrence rate.^3^^,^^9^ While surgical excision led to a successful outcome in this case, medical management may have mitigated symptoms if surgery was not an option. Treatments include continuous combined oral contraceptive pills or gonadotropin releasing hormone agonists to minimize fluctuations in circulating estrogen and progesterone, aiming to achieve more quiescent endometrial tissue.^2^^,^^3^

Although this patient lacked typical symptoms of abdominopelvic endometriosis, her presentation of cutaneous endometriosis prompted further evaluation, including a transvaginal ultrasound, which showed normal results. If symptoms develop in the future, the patient and her parents have been advised to request a complete pelvic workup. Vellido–Cotelo et al (2015) found that in patients with endometriosis identified in a scar, 14% had concomitant pelvic disease.^10^ While associated pelvic endometriosis may be asymptomatic at the time of cutaneous endometriosis diagnosis, it can impact long-term quality of life, contribute to perimenstrual or chronic pelvic pain, and affect fertility. Earlier diagnosis by recognition of cutaneous involvement improved this patient’s knowledge of endometriosis signs and symptoms, which will hopefully improve her overall gynecologic health.

## Conflicts of interest

None disclosed.
